# Prevalence and Factors Associated With Body Pain: Results of a Nationally Representative Survey of 9,586 Chinese Adults Aged 60 and Over

**DOI:** 10.3389/fpubh.2021.634123

**Published:** 2021-03-08

**Authors:** Lu Yang, Wenbo Peng

**Affiliations:** ^1^Faculty of Sociology and Population Sciences, Nanjing University of Posts and Telecommunications, Nanjing, China; ^2^Faculty of Health, Australian Research Centre in Complementary and Integrative Medicine, School of Public Health, University of Technology Sydney, Sydney, NSW, Australia

**Keywords:** pain, older people, China, characterictics, health services research

## Abstract

**Background:** Pain management has become a critical problem worldwide with the aging population. More than half of older people have experienced pain with different severity. The aim of this research is to identify the characteristics of older people with body pain and the associations between pain and characteristics of demographic, health status, and health services use amongst Chinese seniors.

**Methods:** This cross-sectional study was based on the China Health and Retirement Longitudinal Study (CHARLS), using follow-up survey data in 2015. The national survey comprised 20,284 women and men aged 45 years or older who completed questionnaires. Data of older people who were asked whether they had troubles with body pain were extracted and analyzed. Multiple logistic regression modeling was used to determine the important indicators (demographic, health status, and health services use) amongst Chinese elderly with pain.

**Results:** Analyses revealed that 32.5% (*n* = 9,586) of Chinese people aged over 60 reported having body pain. Pain is positively associated with female gender (OR = 2.08, 95% CI 1.80–2.39, *p* < 0.001), living in non-urban areas (OR = 1.49, 95% CI 1.25–1.77, *p* < 0.001), having physical disabilities (OR = 1.45, 95% CI 1.15–1.82, *p* = 0.002), diagnosed with stomach diseases (OR = 1.40, 95% CI 1.20–1.64, *p* < 0.001), diagnosed with arthritis (OR = 1.91, 95% CI 1.66–2.20, *p* < 0.001), self-rating with poor health status (OR = 7.03, 95% CI 5.63–8.78, *p* < 0.001), self-purchased over-the-counter western medications (OR = 1.50, 95% CI 1.30–1.73, *p* < 0.001) and self-purchased Chinese herbal medicine (OR = 1.52, 95% CI 1.24–1.85, *p* < 0.001).

**Conclusion:** Body pain is common amongst the Chinese elderly. This research highlights the need for further nationwide studies exclusively focusing on people with pain including the elder population, and provides evidence-based insights for healthcare providers and policy-makers, to improve the quality of pain management. Future research should also pay attention to the importance of health literacy for health outcomes with regard to pain management.

## Introduction

By 2025, China is projected to have 280 million people aged 60 and over, which is accounted for 20% of the total population (1). Moreover, the proportion of older people will exceed 30% in Europe, North America, Chile, and China by 2050 (2). Population aging has led to increased care needs (3) and overburdened healthcare system (4). China has responded positively to address population aging through a multisectoral approach since the beginning of the 21st century. The recent Healthy China 2030 Action Plan (2019–2030) on aging aims to strengthen older people's health, from the perspective of family, government, and society, making efforts toward decreasing disability rates, providing convenient health services, and establishing home-based care for the older population (5).

Pain has been a global public health issue which is listed as the main cause of disability (6). There are estimates showed that 1 in 5 adults suffer from pain worldwide (7). The prevalence of pain increased with age and issues occurred with regard to the assessment due to cognitive ability changes and socio-cultural factors amongst older people (8). Moreover, body pain is reported to have relation to increased health care use and costs as body pain was associated with significantly more hospitalizations (9, 10) and loss of work productivity (11). A conservative estimate of the economic burden of pain in European countries was USD $185 billion in 2014 (11). Furthermore, health education has been studied to show improvement in terms of pain, disability, and quality of life amongst older people with pain (12).

Given that the pain experienced by older people has a major influence on personal life and social activities (13), there is a need to understand the characteristics of older people with pain in relation to health status and health services use. This information is needed to inform healthcare providers and policy-makers in their decision-making about pain management and the quality of healthcare delivered. However, while higher pain rates among older people have been noticed, information on associations between older people's demographic characteristics, health status, chronic conditions, and health services use are lacking. The aim of the current study is to determine whether body pain is associated with demographic, health, and health services utilization factors in adults aged 60 and over. These findings might contribute to the identification of health strategies for improving health literacy and health outcomes in older people.

## Methods

### Data Source and Analytical Sample

This cross-sectional study was conducted as part of the China Health and Retirement Longitudinal Study (CHARLS), which was designed to examine health and economic adjustments to the rapid aging of the population in China (14). Thirty provincial-level administrative units were selected by stratified random sampling with probabilities proportional to the size to be representative of the general population aged 45 years and over (15). There were 17,708 respondents attracted in the national baseline survey in 2011 and were followed up every 2 years, by using a face-to-face computer-assisted personal interview (CAPI). A detailed description of CHARLS was published elsewhere (14). Data were extracted from the CHARLS in 2015, whereby 2015 is the most current survey data available for this research. For this study, older participants aged 60 and over were analyzed. This study is a secondary analysis of the deidentified CHARLS study data. The original CHARLS was approved by the Ethical Review Committee of Peking University, and all participants signed informed consent at the time of participation.

### Measures of Demographic Characteristics

Older participants were asked about their date of birth, gender, areas of residence (urban or non-urban), current marital status (married/de facto, separated/divorced/widowed/never married), health insurance coverage (including public and private health insurance; no, yes), smoking status (no, yes), and frequency of alcohol use (non-drinker, rarely, often).

### Measures of Health Status and Health Services Use

Participants were asked about their social activity (interacted with friends, played Ma-jong, voluntary work), sports activity (went to a dancing club, gym, practiced Qigong). Information about their self-rated health status (good, fair, poor), physical disability (no, yes) and intellectual disability (no, yes) were also asked. Moreover, the older people were asked about their visits to a range of different healthcare types, including Western Medicine hospitals, specialized hospitals, and Chinese medicine hospitals. Furthermore, the participants were asked whether they used self-treatment methods during the past month, including self-purchased over-the-counter western medicine medications, Chinese herbal medicine, and vitamins/supplements.

### Measures of Chronic Conditions

Participants were questioned whether they had been diagnosed with 14 chronic conditions (hypertension, dyslipidemia, diabetes, cancer, chronic lung disease, liver disease, heart disease, stroke, kidney disease, stomach disease, psychiatric problems, memory-related disease, arthritis, and asthma) by a doctor. Specifically, Alzheimer's disease and dementia were included in the memory-related disease category due to the design of the survey.

### Outcome Measure

Participants responded to the question “Are you often troubled with any body pain?” in the questionnaire and were answered with no or yes. If the participant responded “yes,” they were presented with the question “On what part of your body do you feel pain? (list all body pains).” The parts of body pain include head, shoulder, arm, wrist, fingers, chest, stomach, back, waist, buttocks, legs, knees, ankle, toes, neck, and others.

### Statistical Analysis

Student's *t*-test and χ^2^ tests were used to examine differences in continuously scored and categorical measures, respectively, between older people who had experienced body pain and older people who did not have any troubles with body pain. A multiple logistic regression model was produced to determine the statistically significant factors associated with older people who had experienced with body pains. All the demographic, health status and health services utilization and chronic conditions variables listed above with a *p*-value < 0.2 in the bivariate comparisons or the factors considered clinically important were entered into a model and then a stepwise backward elimination process was employed, to eventually produce the most parsimonious model using a likelihood ratio test. In response to the large sample size, a *p*-value threshold < 0.005 was adopted for statistical significance (16). All analyses were conducted using the statistical programme Stata 16 (StataCorp LP, College Station, Texas, USA).

## Results

The average age of those respondents was 68 years (SD = 6.5), and the majority were females (63.2%). In total, there were 3,117 (32.5%) of 9,586 Chinese aged over 60 having troubles with pain. Amongst those older adults with pain, waist (*n* = 3,906, 68.3%), leg (*n* = 3,020, 52.3%) and knee (*n* = 2,890, 50.5%) were reported to be the top three popular parts of body experiencing pain. A comparison of demographic characteristics between older participants with pain and those without is shown in Table 1. There is no statistically significant difference between those have pain and those without pain in terms of age and insurance status. Older people who had pain were more likely to be females (*P* < 0.001), reside in non-urban areas (*P* < 0.001), being separated/divorced/widowed/never married (*P* < 0.001), being non-drinker than those who did not have troubles with pain.

**Table 1:** Associations between body pain and demographic characteristics, by elderly Chinese.

**Demographic characteristics**		**Pain**		
		**No (*n* = 6,469)**	**Yes (*n* = 3,117)**	***p*-value**
		***n* (%)**	***n* (%)**	
Gender	Female	2,895 (45.3)	1,955 (63.2)	<0.001
	Male	3,493 (54.7)	1,138 (36.8)	
Area of residence	Urban	1,663 (26.3)	505 (16.5)	<0.001
	Non-urban	4,662 (73.7)	2,555 (83.5)	
Marital status	Married/de facto	5,300 (81.9)	2,373 (76.1)	<0.001
	Separated/divorced/widowed/never married	1,168 (18.1)	744 (23.9)	
Insurance status	Yes	5,885 (91.0)	2,806 (90.0)	0.134
Smoking status	Yes	3,219 (49.8)	1,207 (38.7)	<0.001
Alcohol status	Non-drinker	4,192 (64.9)	2,312 (74.2)	<0.001
	Rarely	480 (7.4)	225 (7.2)	
	Often	1,792 (27.7)	578 (18.6)	
		**Mean (SD)**	**Mean (SD)**	
Age		68 (6.7)	68 (6.5)	0.054

Table 2 shows the comparisons between participants who had troubles with pain and those who had not, by health status and health services use. Statistically significant differences were observed amongst older people with pain who had physical disability (*P* < 0.001), intellectual disability (*P* < 0.001), and self-rated general health status (*P* < 0.001). Moreover, compared with older participants without body pain, those with pain were more likely to visit a Western medicine hospital (*P* < 0.001), a specialized hospital (*P* < 0.001), and/or a Chinese medicine hospital (*P* = 0.001). Furthermore, participants who reported self-purchased Chinese herbal medicine (*P* < 0.001) and vitamin/supplements (*P* = 0.001), and over-the-counter western medicine medications (*P* < 0.001) were more likely to have troubles with body pain.

**Table 2:** Associations between body pain and health status and health services use, by elderly Chinese.

**Health status and health services use**		**Pain**		
		**No (*n* = 6,469)**	**Yes (*n* = 3,117)**	***p*-value**
		***n* (%)**	***n* (%)**	
General health status	Good	921 (28.6)	159 (10.5)	<0.001
	Fair	1,901 (59.0)	710 (46.6)	
	Poor	399 (12.4)	652 (42.9)	
Social activity	Yes	3,317 (51.3)	1,426 (45.8)	<0.001
Sport activity	Yes	539 (8.3)	171 (5.5)	<0.001
Intellectual disability	Yes	382 (5.9)	353 (11.3)	<0.001
Physical disability	Yes	472 (7.3)	408 (13.1)	<0.001
Western Medicine hospital	Yes	413 (6.4)	328 (10.5)	<0.001
Specialized hospital	Yes	39 (0.6)	42 (1.3)	<0.001
Chinese medicine hospital	Yes	76 (1.2)	63 (2.0)	0.001
Self-Purchased over-the-counter western medicine medications	Yes	1,974 (30.5)	1,366 (43.8)	<0.001
Self-purchased traditional herbal medicine	Yes	634 (9.8)	565 (18.1)	<0.001
Self-purchased Vitamins/supplements	Yes	561 (8.7)	339 (10.9)	0.001

Table 3 shows the associations between older participants' chronic conditions and their status of body pain. Statistically significant differences were observed amongst older people with pain and clinically important variables of health conditions. Specifically, compared with those participants who did not have body pain, older participants who had troubles with pain were more likely to have hypertension (*P* < 0.001), dyslipidemia (*P* = 0.004), diabetes (*P* = 0.001), chronic lung diseases (*P* < 0.001), liver diseases (*P* < 0.001), stroke (*P* < 0.001), kidney diseases (*P* < 0.001), stomach diseases (*P* < 0.001), psychiatric problems (*P* = 0.001), memory-related diseases (*P* < 0.001), arthritis (*P* < 0.001) and/or asthma (*P* < 0.001).

**Table 3:** Associations between body pain and chronic conditions, by elderly Chinese.

**Chronic conditions**		**Pain**		
		**No (*n* = 6,469)**	**Yes (*n* = 3,117)**	***p*-value**
		***n* (%)**	***n* (%)**	
Hypertension	Yes	1,730 (26.7)	999 (32.0)	<0.001
Dyslipidemia	Yes	690 (10.7)	395 (12.7)	0.004
Diabetes	Yes	428 (6.6)	267 (8.6)	0.001
Cancer	Yes	69 (1.1)	40 (1.3)	0.349
Chronic lung diseases	Yes	726 (11.2)	545 (17.5)	<0.001
Liver diseases	Yes	235 (3.6)	184 (5.9)	<0.001
Heart diseases	Yes	828 (12.8)	619 (19.9)	<0.001
Stroke	Yes	165 (2.5)	121 (3.9)	<0.001
Kidney diseases	Yes	346 (5.4)	339 (10.9)	<0.001
Stomach diseases	Yes	1,334 (20.6)	1,095 (35.1)	<0.001
Psychiatric problems	Yes	72 (1.1)	60 (1.9)	0.001
Memory-related diseases	Yes	105 (1.6)	106 (3.4)	<0.001
Arthritis	Yes	2,086 (32.2)	1,777 (57.0)	<0.001
Asthma	Yes	272 (4.2)	228 (7.3)	<0.001

*Memory-related diseases include Alzheimer's disease and dementia*.

The statistically significant predictors of body pain amongst older participants, as determined by logistic regression modeling, are presented in Table 4. The Hosmer and Lemeshow goodness-of-fit statistic for this regression model was insignificant (*R*^2^ = 16.1%, *P* = 0.340), showing the percentage of variation of older people who had experienced with body pains described by this model was 16.1%. Older participants who were female had higher odds (OR = 2.08, 95% CI 1.80–2.39, *p* < 0.001) of reporting pain compared with those who were male. Older people who reported their general health status being fair (OR = 1.77, 95% CI 1.45–2.16, *p* < 0.001) or poor (OR = 7.03, 95% CI 5.63–8.78, *p* < 0.001) had higher odds of having troubles with pain compared with those who rated their general health status being good. Older participants who resided in non-urban areas (OR = 1.49, 95% CI 1.25–1.77, *p* < 0.001), having physical disabilities (OR = 1.45, 95% CI 1.15–1.82, *p* = 0.002), diagnosed with arthritis (OR = 1.91, 95% CI 1.66–2.20, *p* < 0.001) and/or stomach diseases (OR = 1.40, 95% CI 1.20–1.64, *p* < 0.001) had higher odds of reporting pain compared with those who resided in urban areas, having no physical disabilities and no stomach diseases. Furthermore, older participants who purchased over-the-counter western medicine medications (OR = 1.50, 95% CI 1.30–1.73, *p* < 0.001) or used Chinese herbal medicine (OR = 1.52, 95% CI 1.24–1.85, *p* < 0.001) were more likely to have troubles with body pain.

**Table 4:** Logistic regression identifying the statistically significant predictors of body pain by the Chinese elderly.

**Predictors of pain**		**Odds ratio**	**95% CI**	***p*-value**
Gender	Male	1.00	–	<0.001
	Female	2.08	1.80, 2.39	
General health status	Good	1.00	–	<0.001
	Fair	1.72	1.45, 2.16	
	Poor	7.03	5.63, 8.78	
Area of Residence	Urban	1.00	–	<0.001
	Non-urban	1.49	1.25, 1.77	
Physical disability	No	1.00	–	0.002
	Yes	1.45	1.15, 1.82	
Arthritis	No	1.00	–	<0.001
	Yes	1.91	1.66, 2.20	
Stomach diseases	No	1.00	–	<0.001
	Yes	1.40	1.20, 1.64	
Purchased over-the-counter western medications	No	1.00	–	<0.001
	Yes	1.50	1.30, 1.73	
Traditional Chinese herbal medicine	No	1.00	–	<0.001
	Yes	1.52	1.24, 1.85	

## Discussion

This study presents the evidence of a large-scale population study exploring health status, chronic conditions, and health services use amongst Chinese people aged over 60 years old with pain. There were 32.5% of older participants experienced pain in this study and older women were more likely to experience body pain compared to older men. These findings are in accordance with previous population studies (17, 18), suggesting aging increased on pain prevalence and women might be more sensitive to the feeling of pain (18). However, the prevalence of pain in this study is lower than a cross-sectional cohort research, indicating 48.5% of 45,418 adults aged over 65 years reported experiencing daily pain in New Zealand (19). The variation in the prevalence of body pain could be partially explained by racial/ethnical and cultural factors.

This study found that self-rated health status was related to body pain, and it has been noted that the poorer self-rated health, the more likely for those older people to have body pain. The results are consistent with the study on 28,800 middle-aged and older Canadians, which showed that the odds of reporting good self-rated health are 4–5 times lower for those with pain, compare to the odds of reporting good self-rated health among those without pain (20). Moreover, this finding from the current research is in line with findings from a recent longitudinal study of 7,523 Norwegian, indicating individuals who shift from low back pain symptoms to remission of symptoms are more likely to have better self-rated health (21). Thus, this research lends further support to the notion that pain management or prevention may have significant potential in improving self-rated health amongst the older population (22). Further studies on pain management or pain education are warranted.

The results from this research showed that chronic conditions such as stomach disease and/or arthritis are the predictors of older people reporting body pain. There is no doubt that arthritis, along with other chronic non-communicable diseases (NCDs) is positively correlated with chronic pain (7), one of its various symptoms. Moreover, research has shown greater pain disability (the degree to which chronic pain interferes with daily activities) experienced lower disease self-efficacy amongst 141 older adults with arthritis (23). Given that NCDs are estimated to be accounted for 80% of the global burden of disease in 2020 (24), NCDs-related pain relief interventions are needed to relieve suffering for millions of NCDs' patients, as well as strengthen health systems, especially for those in developing countries. Understanding the relationships between body pain and chronic conditions may be helpful to relieve symptoms of those patients with NCDs and consider pain as part of the NCDs' management strategy in order to improve their quality of life.

A link between the use of Chinese herbal medicine and body pain has previously been reported (25). This study found that older people with body pain were 1.52 times more likely to self-purchase traditional herbal medicine. A possible explanation for this finding could be the fact that systematic reviews and meta-analyses of Chinese herbal medicine for pain management have indicated positive effect amongst older people (26, 27). Moreover, research has shown that older people were more likely to try complementary medicine (including Chinese herbal medicine) for treating pain symptoms (28). However, the majority of TCM utilization by people with pain is concurrent to conventional medications (29) and this raises the potential of a number of direct and indirect risks. A more sophisticated level of detail about how, when, and why older people with pain use Chinese herbal medicine and further empirical work on this topic is required to help inform conventional health practitioners, policy-makers, and patients to ensure safe, effective coordinated care for those with pain.

There are several limitations to this study. The interpretation of these findings is limited by the fact that data came from a cross-sectional study which could only examine the status of those older people with body pain at the same point in time. Moreover, the body pain in this research was not assessed by a validated, subjective measure for pain but based on respondents' self-reported details. Self-reported information by older participants may be open to recall bias. In addition, the study sample was restricted to participants aged over 60 who responded to pain-related questions and there were 8,649 participants aged 45–60 who had pain but not analyzed, as such, results from this research may not be generalisable to all older adult Chinese women and men. Furthermore, there may be confounders in addition to those reported in this study that contribute to pain status amongst older people (e.g., frequency, intensity, and period of pain). Nevertheless, these limitations are countered by the fact that this study is a secondary data analyses from a comprehensively nationally representative sample in an established and large dataset, which provides an analysis of health status and health services use with regard to older people with pain.

## Conclusions

This paper was designed to provide the first comprehensive determinants of characteristics of demographic, health status, and health services use amongst Chinese seniors with pain from a public health perspective and hope to help clinicians encourage their older patients with pain to disclose such information to evaluate pain. Moreover, the results from this research provide evidence-based insights for policy-makers to improve the quality of pain management. Further research is needed to assess older people's pain perception in order to improve health literacy amongst those people with pain.

## Data Availability Statement

Publicly available datasets were analyzed in this study. This data can be found here: http://charls.pku.edu.cn/pages/data/2015-charls-wave4/zh-cn.html.

## Author Contributions

LY drafted the manuscript. WP contributed to design and revision. All authors contributed to the article and approved the submitted version.

## Conflict of Interest

The authors declare that the research was conducted in the absence of any commercial or financial relationships that could be construed as a potential conflict of interest.

## References

[1] Mao G Lu F Fan X Wu D. China's Ageing Population: The Present Situation and Prospects. Population Change and Impacts in Asia and the Pacific. Singapore: Springer (2020). p. 269–87.

[2] World Health Organization. World Report on Ageing and Health. Luxemburg: World Health Organization (2015).

[3] Kingston A Comas-Herrera A Jagger C. Forecasting the care needs of the older population in England over the next 20 years: estimates from the population ageing and care simulation (PACSim) modelling study. Lancet Public Health. (2018) 3:e447–55. 10.1016/S2468-2667(18)30118-X 30174210 PMC6123499

[4] Wang X-Q Chen P-J. Population ageing challenges health care in China. Lancet. (2014) 383:870. 10.1016/S0140-6736(14)60443-8 24607099

[5] National Health Commission of the People's Republic of China. Healthy China Action Plan (2019-2030). Beijing: National Health Commission of the People's Republic of China (2019).

[6] Yang G Wang Y Zeng Y Gao GF Liang X Zhou M. Rapid health transition in China, 1990–2010: findings from the global burden of disease study 2010. Lancet. (2013) 381:1987–2015. 10.1016/S0140-6736(13)61097-1 23746901 PMC7159289

[7] Goldberg DS McGee SJ. Pain as a global public health priority. BMC Public Health. (2011) 11:1–5. 10.1186/1471-2458-11-770 21978149 PMC3201926

[8] Schofield P. The assessment of pain in older people: UK national guidelines. Age Ageing. (2018) 47 (suppl_1):i1–i22. 10.1093/ageing/afx192 29579142 PMC5888957

[9] Hadjiat Y Serrie A Treves R Chomier B Geranton L Billon S. Pain associated with health and economic burden in France: results from recent national health and wellness survey data. Clin Econ Outcomes Res. (2018) 10:53. 10.2147/CEOR.S148405 29403296 PMC5779295

[10] Antaky E Lalonde L Schnitzer ME Martin É Berbiche D Perreault S. Identifying heavy health care users among primary care patients with chronic non-cancer pain. Can J Pain. (2017) 1:22–36. 10.1080/24740527.2017.1326088 PMC8730606 35005339

[11] Witt EA Kenworthy J Isherwood G Dunlop WC. Examining the association between pain severity and quality-of-life, work-productivity loss, and healthcare resource use among European adults diagnosed with pain. J Med Econ. (2016) 19:858–65. 10.1080/13696998.2016.1178127 27074532

[12] Zahari Z Ishak A Justine M. The effectiveness of patient education in improving pain, disability and quality of life among older people with low back pain: a systematic review. J Back Musculoskelet Rehabil. (2020) 33:245–54. 10.3233/BMR-181305 31356191

[13] Xu X Li B Liu L Zhao Y. Body pain intensity and interference in adults (45–53 years old): a cross-sectional survey in Chongqing, China. Int J Environ Res Public Health. (2016) 13:887. 10.3390/ijerph13090887 27618073 PMC5036720

[14] Zhao Y Hu Y Smith J Strauss J Yang G. Cohort profile: the China health and retirement longitudinal study (CHARLS). Int J Epidemiol. (2014) 43:61–8. 10.1093/ije/dys203 23243115 PMC3937970

[15] Liu N Cadilhac D Kilkenny M Liang Y. Changes in the prevalence of chronic disability in China: evidence from the China health and retirement longitudinal study. Public Health. (2020) 185:102–9. 10.1016/j.puhe.2020.03.032 32603874

[16] Glymour C Madigan D Pregibon D Smyth P. Statistical themes and lessons for data mining. Data Mining Knowledge Discov. (1997) 1:11–28. 10.1023/A:1009773905005

[17] Raggi A Leonardi M Mellor-Marsá B Moneta MV Sanchez-Niubo A Tyrovolas S. Predictors of pain in general ageing populations: results from a multi-country analysis based on ATHLOS harmonized database. J Headache Pain. (2020) 21:1–12. 10.1186/s10194-020-01116-3 32375641 PMC7201730

[18] Tong Y Jun M Yan JIANG JL Yunlong Gen YW Wenjie S. Assessing pain among Chinese elderly-chinese health and retirement longitudinal study. Iran J Public Health. (2018) 47:553. 29900140 PMC5996333

[19] Crowe M Jordan J Gillon D McCall C Frampton C Jamieson H. The prevalence of pain and its relationship to falls, fatigue, and depression in a cohort of older people living in the community. J Adv Nurs. (2017) 73:2642–51. 10.1111/jan.13328 28475222

[20] Chireh B D'Arcy C. Pain and self-rated health among middle-aged and older Canadians: an analysis of the Canadian community health survey—healthy aging. BMC Public Health. (2018) 18:1006. 10.1186/s12889-018-5912-9 30103705 PMC6090606

[21] Nordstoga AL Nilsen TIL Vasseljen O Unsgaard-Tøndel M Mork PJ. The influence of multisite pain and psychological comorbidity on prognosis of chronic low back pain: longitudinal data from the Norwegian HUNT study. BMJ Open. (2017) 7:e015312. 10.1136/bmjopen-2016-015312 28592580 PMC5734202

[22] Karjalainen M Tiihonen M Kautiainen H Saltevo J Haanpää M Mäntyselkä P. Pain and self-rated health in older people with and without type 2 diabetes. Eur Geriatr Med. (2018) 9:127–31. 10.1007/s41999-017-0017-z 34654280

[23] James NT Miller CW Brown KC Weaver M. Pain disability among older adults with arthritis. J Aging Health. (2005) 17:56–69. 10.1177/0898264304272783 15601783

[24] Islam SMS Purnat TD Phuong NTA Mwingira U Schacht K Fröschl G. Non-communicable diseases (NCDs) in developing countries: a symposium report. Global Health. (2014) 10:1–8. 10.1186/s12992-014-0081-9 25498459 PMC4267750

[25] Yuan Q-l Guo T-m Liu L Sun F Zhang Y-g. Traditional Chinese medicine for neck pain and low back pain: a systematic review and meta-analysis. PLoS ONE. (2015) 10:e0117146. 10.1371/journal.pone.0117146 25710765 PMC4339195

[26] Lin X Huang K Zhu G Huang Z Qin A Fan S. The effects of acupuncture on chronic knee pain due to osteoarthritis: a meta-analysis. J Bone Joint Surg Am Vol. (2016) 98:1578–85. 10.2106/JBJS.15.00620 27655986

[27] Chen B Zhan H Marszalek J Chung M Lin X Zhang M. Traditional Chinese medications for knee osteoarthritis pain: a meta-analysis of randomized controlled trials. Am J Chin Med. (2016) 44:677–703. 10.1142/S0192415X16500373 27222066 PMC5553612

[28] Chen FP Chen TJ Kung YY Chen YC Chou LF Chen FJ. Use frequency of traditional Chinese medicine in Taiwan. BMC Health Serv Res. (2007) 7:26. 10.1186/1472-6963-7-26 17319950 PMC1810531

[29] Chen M-C Lai J-N Chen P-C Wang J-D. Concurrent use of conventional drugs with Chinese herbal products in Taiwan: a population-based study. J Trad Complement Med. (2013) 3:256–62. 10.4103/2225-4110.119734 24716186 PMC3925000

